# Modeling pathogenesis of emergent and pre-emergent human coronaviruses in mice

**DOI:** 10.1007/s00335-018-9760-9

**Published:** 2018-07-24

**Authors:** Adam S. Cockrell, Sarah R. Leist, Madeline G. Douglas, Ralph S. Baric

**Affiliations:** 1 Department of Epidemiology, University of North Carolina-Chapel Hill, Chapel Hill, NC 27599 USA; 2 Department of Microbiology and Immunology, University of North Carolina-Chapel Hill, Chapel Hill, NC 27599 USA

## Abstract

The emergence of highly pathogenic human coronaviruses (hCoVs) in the last two decades has illuminated their potential to cause high morbidity and mortality in human populations and disrupt global economies. Global pandemic concerns stem from their high mortality rates, capacity for human-to-human spread by respiratory transmission, and complete lack of approved therapeutic countermeasures. Limiting disease may require the development of virus-directed and host-directed therapeutic strategies due to the acute etiology of hCoV infections. Therefore, understanding how hCoV–host interactions cause pathogenic outcomes relies upon mammalian models that closely recapitulate the pathogenesis of hCoVs in humans. Pragmatism has largely been the driving force underpinning mice as highly effective mammalian models for elucidating hCoV–host interactions that govern pathogenesis. Notably, tractable mouse genetics combined with hCoV reverse genetic systems has afforded the concomitant manipulation of virus and host genetics to evaluate virus–host interaction networks in disease. In addition to assessing etiologies of known hCoVs, mouse models have clinically predictive value as tools to appraise potential disease phenotypes associated with pre-emergent CoVs. Knowledge of CoV pathogenic potential before it crosses the species barrier into the human population provides a highly desirable preclinical platform for addressing global pathogen preparedness, an overarching directive of the World Health Organization. Although we recognize that results obtained in robust mouse models require evaluation in non-human primates, we focus this review on the current state of hCoV mouse models, their use as tractable complex genetic organisms for untangling complex hCoV–host interactions, and as pathogenesis models for preclinical evaluation of novel therapeutic interventions.

## Introduction

The etiologies of coronaviruses (CoVs) comprise an extensive host range that includes reptiles, birds, pigs, dogs, cats, cattle, rodents, bats, camels, and humans. The extensive non-human host range may serve as reservoirs for pre-emergent coronaviruses poised for zoonotic transmission into human and animal populations (Forni et al. 2017; Zhou et al. 2018). There are six known human coronaviruses (OC43, 229E, NL-63, HKU1, SARS-CoV, and MERS-CoV), four of which (NL-63, HKU1, SARS-CoV, and MERS-CoV) were identified in the last 15 years. Considering that bats may be the pinnacle reservoir for the origin of most hCoVs (SARS-CoV, MERS-CoV, 229E, and NL63), evidence for SARS-CoV and MERS-CoV indicates that intermediate zoonotic hosts (civet cat and camel, respectively) may be as important for the evolution of a pathogenic CoV to emerge into the human population (Forni et al. 2017). The emergence of novel pathogenic CoVs into the human population can result in highly lethal respiratory hCoVs with global pandemic potential through human-to-human spread, which is underscored by the global spread of SARS-CoV (~ 10% mortality) in 2002–2003 and the ongoing re-introduction of MERS-CoV (~ 35% mortality) into humans on the Arabian Peninsula. Human-to-human transmission of MERS-CoV was most apparent in the South Korean outbreak in 2015, established by a single individual returning from a visit to the Arabian Peninsula, and resulting in 186 individuals infected with an ~ 20% mortality rate (Lee 2015). The high mortality rate in South Korea also established a society-wide panic that led to a severe economic toll, anticipated to cut 0.1% from the GDP rate in 2015 (Lee 2015). Once an hCoV emerges in humans new tools and robust mouse models are needed to decipher pathogenic mechanisms. Ultimately, understanding the hCoV–host molecular interaction networks that regulate pathogenesis can guide the engineering of therapeutic countermeasures.

**Fig. 1:**
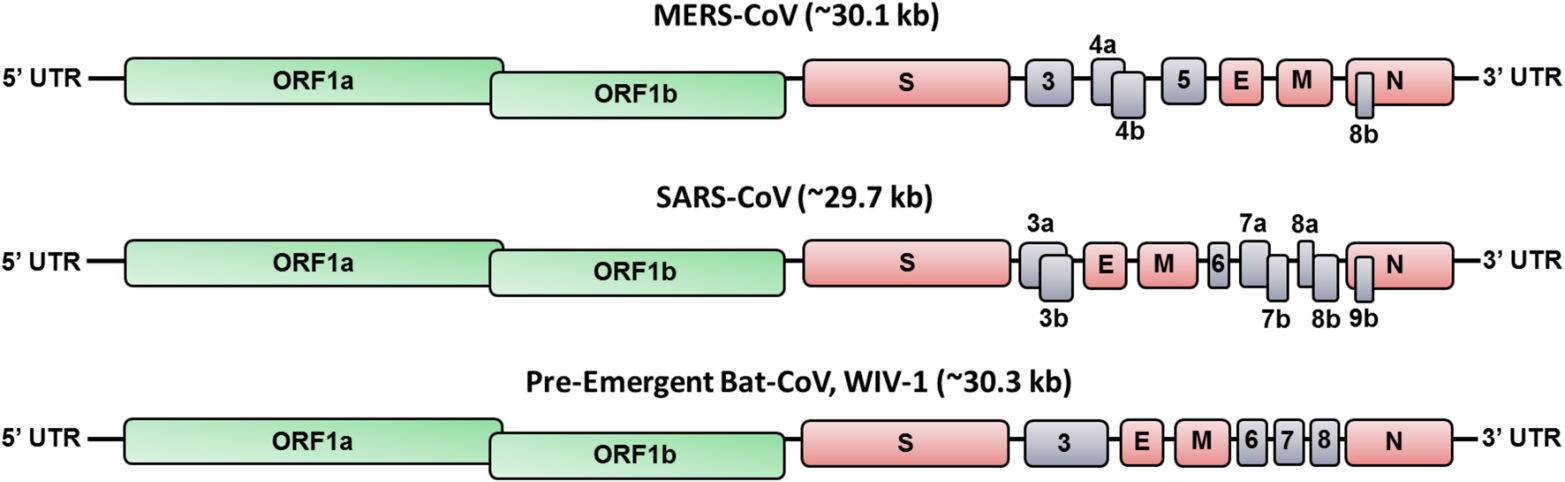
Emergent and pre-emergent coronavirus genome organization. The *Orf1a* and *Orf1b* genes (green) encode 16 non-structural proteins (nsp1–nsp16) that are highly conserved throughout coronaviruses. The structural genes (red) encode the structural proteins spike (S), envelope (E), membrane (M), and nucleocapsid (N), which are common to all coronaviruses. The accessory genes (dark shade) are unique to different coronaviruses with regard to number, genomic organization, sequence, and function

**Fig. 2:**
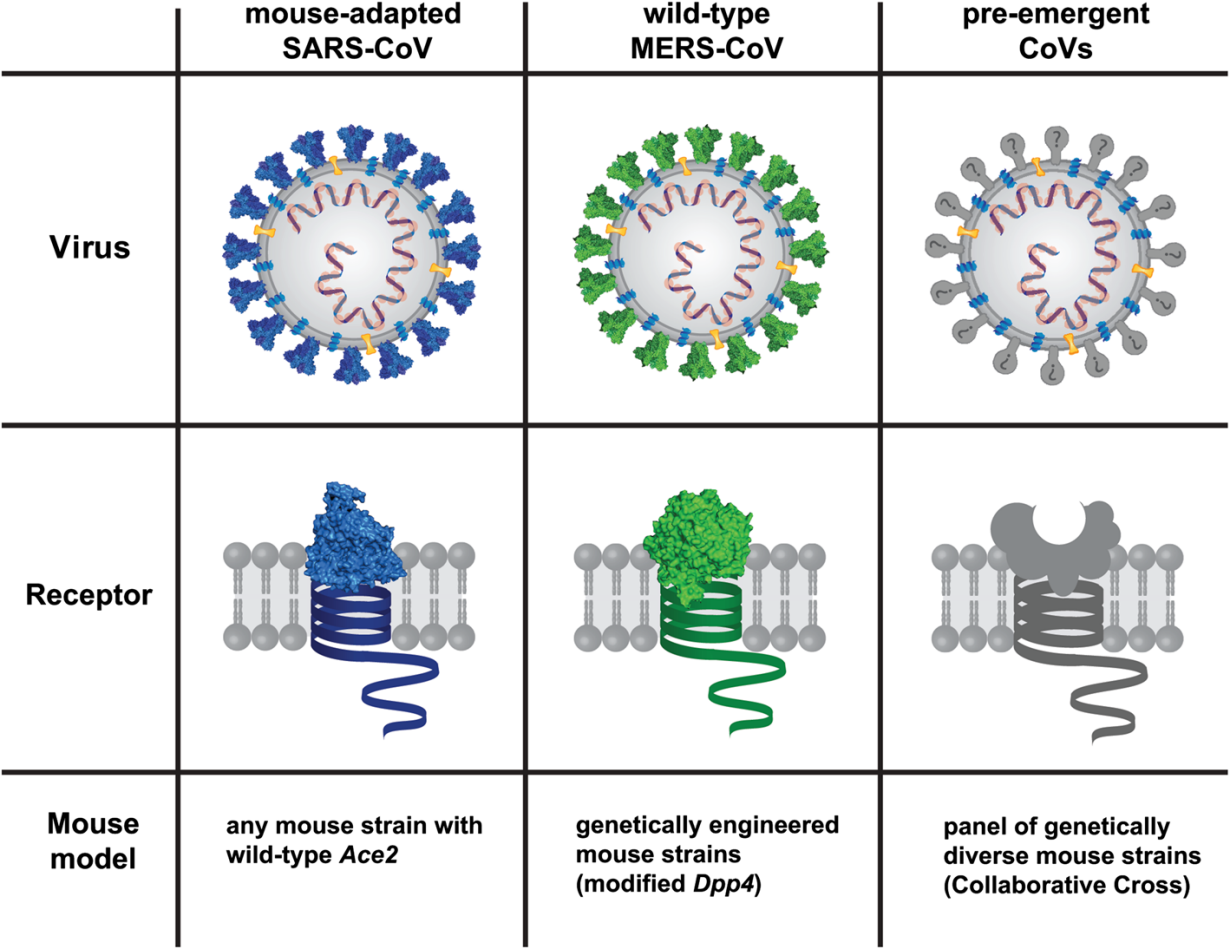
Mouse model development should be tailored to the coronavirus of interest. *Mouse-adapted SARS-CoV column (blue)*. SARS-CoV infects humans via an interaction of the spike protein [RCSB PDB ID: 5X58 (Yuan et al. 2017)] with its cognate receptor, ACE2 [RCBS PDB ID: 1R42 (Towler et al. 2004)]. In order to establish lethal mouse models, the spike protein, and other genomic determinants, have been modified through adaptive evolution in mouse lungs (Day et al. 2009; Frieman et al. 2012; Roberts et al. 2007a). Thereby, any mouse subspecies that exhibits an unaltered mouse *Ace2* can be infected with the mouse-adapted SARS-CoV. *Wild-type MERS-CoV column (green)*. MERS-CoV infects humans via an interaction of the spike protein [RCSB PDB ID: 5X5F (Yuan et al. 2017)] with its cognate receptor, DPP4 [RCSB PDB ID: 2ONC (Feng et al. 2007)]. The MERS-CoV spike protein is not able to interact with the mouse orthologue of human *DPP4* (Cockrell et al. 2014; Coleman et al. 2014). Therefore, the mouse *Dpp4* gene had to be genetically modified in order to allow for infection with MERS-CoV (Cockrell et al. 2016; Li et al. 2017; Pascal et al. 2015). *Pre-emergent CoVs column (grey)*. Pre-emergent CoVs can use either known human receptors for CoVs (ACE2 or DPP4) (Ge et al. 2013; Menachery et al. 2015; Wang et al. 2014; Yang et al. 2016, 2014), or novel, unknown receptors to infect their host. The genetically highly diverse panel of mouse strains from the Collaborative Cross has the potential to provide mouse models for studies of newly emerging CoVs due to the high genetic variability present in this resource. For all virus images: yellow represents the envelope protein; light/dark blue represents the membrane protein; and red represents the nucleocapsid protein

An ideal strategy for development of models that recapitulate human respiratory CoVs clearly includes the development of a genetically tractable mammalian model that effectively recapitulates human pathogenic phenotypes, and an efficient reverse genetics system for seamless manipulation of the hCoV genome. Genetic control of both host and hCoV genomes facilitates the ability to draw cause-and-effect relationships between virus/host genetics and pathogenic outcomes. Reverse systems genetic tools were developed to manipulate the large hCoV genomes (~ 30 kb) that encode 16 non-structural proteins; structural proteins including spike (S), envelope (E), membrane (M), and nucleocapsid (N); and a number of accessory proteins that vary in sequence and function (Fig. 1) [reviewed in (Cockrell et al. 2017)]. A number of in vitro experiments (biochemical and tissue culture based) demonstrated that hCoV proteins interact with host cell proteins to ensure fidelity during virus replication or facilitate evasion of host cell immune responses. The most prominent hCoV–host cell interaction is between the major determinant of viral tropism, the spike protein, and a host cell-specific receptor. The spike protein forms a trimer on the surface of the virus with each monomer harboring a receptor binding domain (RBD) that interacts with its cognate receptor on the host cell (ACE2 for SARS-CoV and DPP4 for MERS-CoV) (Fig. 2). The spike protein, especially the RBD, is a major target for development of antibody and vaccine therapeutics. Other highly conserved structural and non-structural proteins, chiefly enzymatic proteins, may serve as targets for broadly effective anti-hCoV therapeutic targets. The accessory proteins are not conserved between hCoVs, and numerous studies revealed that these gene sets encode critical determinants of species-specific pathogenesis by modulating host immune responses (Fig. 1). In vitro studies have shown a number of accessory proteins to be important for evasion of host innate immune responses through interactions with host proteins [reviewed in (de Wit et al. 2016; Totura and Baric 2012)]. Although in vitro studies are imperative for understanding molecular and biochemical mechanisms, only in vivo studies can decipher how complex hCoV–host interactions relate to pathogenic phenotypes following a respiratory infection.

Discerning the fine balance between pathogenesis and protection, that is governed by the relationship between host innate and adaptive immune responses, requires the development of mammalian models that effectively recapitulate the pathogenesis of respiratory hCoVs. The challenges associated with modeling pathogenesis of emergent and pre-emergent hCoVs are borne out of models developed for SARS-CoV and MERS-CoV over the last 15 years (Table 1; Fig. 2). Mice, ferrets, and non-human primates (NHPs) are traditionally used for the studies involving respiratory pathogens and are among the species initially investigated for SARS-CoV and MERS-CoV replication and pathogenesis in the lungs. Taking into account a number of practical considerations when comparing models for both SARS-CoV and MERS-CoV, mouse models appear to be the most pragmatic (Table 1). Although we emphasize the practicality of hCoV mouse models for the purposes of this review and the absolute critical need for robust primate models for downstream drug and vaccine testing, it is important to note that research (e.g., efficacy of therapeutic countermeasures) established in a robust mouse model should effectively translate to large animal models, such as NHPs, that more closely reflect human physiology.

**Table 1:**
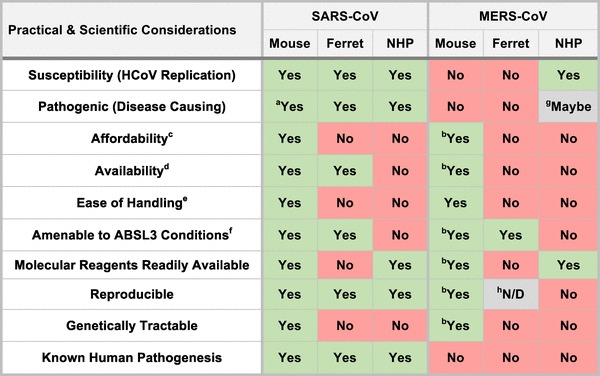
Practical considerations for establishing an effective HCoV animal model

^a^Yes—required virus adaptation

^b^Yes—after genetically engineering a mouse

^c^Affordability—more cost effective relative to ferret and NHP models

^d^Availability—developed models that can be readily acquired and studied by the broader scientific community

^e^Ease of handling—mice require least amount of specialized training compared to ferrets and NHPs

^f^Amenable to ABSL3 conditions—mice are most amenable to ABSL3 conditions due to space limitations, specialized handling requirements, and personnel limitations

^g^Maybe—dependent on species and techniques

^h^N/D—not determined

In general, mouse models for both SARS-CoV and MERS-CoV are affordable, readily available, require nominal training for handling, amenable to manipulation under BSL3 conditions, amenable to analysis with commercially available molecular reagents, amenable to large numbers for purposes of experimental reproducibility, and are genetically tractable (Table 1). Despite these commonalities, establishing a mouse model for MERS-CoV presented challenges not encountered with SARS-CoV (Table 1). Even now, 6 years after the first case of MERS-CoV, one of the biggest challenges remains a very limited understanding of the pathology of MERS-CoV in humans. Only two studies have described the pathology of MERS-CoV in two fatal human cases (Alsaad et al. 2017; Ng et al. 2016). The two fatal human MERS-CoV cases were similar to those observed in cases of SARS-CoV, wherein patients exhibited severe acute respiratory distress with diffuse alveolar damage and formation of hyaline membranes, alveolar fibrin deposits, edema, hemorrhaging, cellular debris, alveolar and interstitial inflammation throughout lungs, and extensive pulmonary tissue necrosis (Alsaad et al. 2017; Ng et al. 2016). Fatal cases of SARS-CoV and MERS-CoV were commonly associated with a gradual loss of respiratory function with available intervention restricted to mechanical respiratory support and oxygen supplementation [reviewed in (Arabi et al. 2017)]. Therefore, effective mouse models for SARS-CoV and MERS-CoV should minimally be able to recapitulate fatal respiratory disease having pathology similar to that observed in humans. As described below, a number of mouse models exhibiting fatal respiratory disease were developed for SARS-CoV and MERS-CoV; however, a single impediment was realized early in model development for MERS-CoV that was not confronted for SARS-CoV. The mouse orthologue of the human receptor for MERS-CoV, dipeptidyl peptidase 4 (DPP4), did not support interaction with the MERS-CoV spike glycoprotein RBD (Cockrell et al. 2014). Therefore, unlike SARS-CoV, commercially available mice were not susceptible to MERS-CoV infection and replication (Coleman et al. 2014). Susceptibility and MERS-CoV pathogenesis was achieved using unique approaches to genetically engineer mice, as described in detail below. Establishing mouse models of fatal disease for SARS-CoV and MERS-CoV has afforded the necessary knowledge to institute a preclinical in vivo platform that can be used to evaluate pre-emergent hCoV respiratory pathogens with unknown disease outcomes (Fig. 2). The strategy is widely portable to other important human pathogens that replicate poorly or not at all in the mouse.

### Mouse models for SARS-CoV pathogenesis

The need for SARS-CoV mouse models was recognized shortly after the SARS-CoV outbreak that spread globally in 2002–2003. Three different strategies were employed for development of SARS-CoV mouse models: (i) different mouse species (or subspecies) were challenged with wild-type human SARS-CoV isolates in order to find a model that allows for replication and reflects severe respiratory disease symptoms observed in infected human patients; (ii) mice were genetically engineered to modify the host receptor, which facilitated productive SARS-CoV replication and pathogenesis; and (iii) adaptive evolution of wild-type SARS-CoV to a chosen mouse species was done to enhance pathogenesis, and associated clinical phenotypes in vivo.

Initial studies to develop a mouse model for SARS-CoV evaluated the susceptibility of various mouse subspecies to clinical isolates of SARS-CoV. In early 2004, the first study using the mouse as a model organism for SARS-CoV was reported (Subbarao et al. 2004). 4–6 weeks old female BALB/c mice were intra-nasally infected with a clinical isolate of SARS-CoV (Urbani) and efficient viral replication was observed from the upper and lower respiratory tract with peak titers on day 3 and clearance by day 7, whereas viral replication was not detected in other organs. However, mice continued to gain weight and did not show other signs of clinical disease. Passive transfer of immune sera to naïve mice was sufficient to prevent viral replication in the respiratory tract, indicating that wild-type SARS-CoV could elicit an effective humoral immune response. Nonetheless, in the absence of pathogenesis with no clinical signs of disease, it is difficult to properly evaluate the efficacy of therapeutic countermeasures. In a second study, Glass et al. used C57BL/6 mice which were known to exhibit a Th1-biased immune response in contrast to BALB/c mice that were considered to be Th2-biased (Glass et al. 2004), which may account for differences in pathogenic outcomes. Like the BALB/c study, 5–6 weeks old C57BL/6 mice were infected with the clinical SARS-CoV Urbani isolate. Infected C57BL/6 mice exhibited slower weight gain than mock-infected mice and SARS-CoV was found not only in the upper and lower respiratory tract but also in the brain, which was not associated with clinical disease in humans. Developing a SARS-CoV model in C57BL/6 mice had the added benefit of expanding the applicability of the model to genetically modified knock-out (KO) mice that are predominantly generated on a C57BL/6 background, thereby facilitating the investigation of host genetics on SARS-CoV pathogenesis (Glass et al. 2004). Utilizing *CD-1*^−/−^ and *Rag*^−/−^ knock-out mice, Glass et al. demonstrated that NK cells, NK-T cells, or T and B lymphocytes are not required for clearance of SARS-CoV in the mouse (Glass et al. 2004). These initial studies to evaluate different murine subspecies as models for SARS-CoV resulted in replication in the respiratory tract with no indication of the clinical pathogenesis often observed in human cases experiencing severe respiratory distress.

Knock-out mice deficient in the innate immune response were the first to exhibit clinical alterations that included weight loss and progressively worsening pulmonary disease (Hogan et al. 2004). 129SvEv and *Stat1*^−/−^ mice on a 129SvEv background infected with a SARS-CoV clinical isolate (Toronto-2) showed that *Stat1* was required for the resolution of SARS-CoV infection. Clinical signs of disease were only observed in KO mice, whereas efficient viral replication in the lower respiratory tract caused no clinical symptoms in 129SvEv wild-type mice. In contrast, *Stat1*^−/−^ mice supported viral replication with peak titers on day 3 and persistence throughout day 22 (Hogan et al. 2004). Most features of pathology that were found in *Stat1*^−/−^ mice were different from those found in humans. However, some pathological features common to humans, such as bronchiolar injury with focal respiratory epithelial cell necrosis, supported the idea that the mouse species could be a good model for SARS-CoV-associated clinical findings observed in the respiratory tract of infected human patients.

Increased fatality as consequence of acute respiratory distress syndrome (ARDS) from SARS-CoV infection was often associated with advanced age (> 60 years old) in humans (Booth et al. 2003; Donnelly et al. 2003; Tsui et al. 2003), which was exploited to improve mouse models of SARS-CoV pathogenesis. To replicate advanced age in humans, 12–14 months old BALB/c (Roberts et al. 2005), C57BL/6, and 129S6 (Roberts et al. 2008) mice were intra-nasally infected with a clinical isolate of SARS-CoV (Urbani). BALB/c and C57BL/6 mice showed comparable levels and kinetics of viral replication while aged 129S6 mice cleared SARS-CoV by day 5 (Roberts et al. 2008). The predominant clinical feature regarding mouse studies is weight loss, a consequence of appetite loss and dehydration. All three lines of aged mice showed transient signs of clinical disease including weight loss, ruffled fur, hunching, and dehydration that resolved by day 7. Moreover, all three strains exhibited histopathological findings such as perivascular and peribronchiolar infiltrates, necrotic debris in bronchioles, and interstitial pneumonitis. Importantly, extensive alveolar damage in aged BALB/c mice persisted through day 9 post-infection (p.i.), findings that closely resembled pathology observed in humans. These are some of the first controlled studies to demonstrate that differences in host genetics can significantly influence SARS-CoV pathogenesis, a topic that is revisited below with regard to using a tractable population of genetically distinct mice referred to as the Collaborative Cross. Concurrent with these studies, Rockx et al. demonstrated that pathogenesis related to SARS-CoV clinical isolates was also dependent upon the genetics of the SARS-CoV strain evaluated in vivo (Rockx et al. 2007). Using reverse genetics Rockx et al. modified the spike protein from the SARS-CoV Urbani strain to encode the spike from middle (CUHK-W1) and early (GZ02) phase clinical isolates or a civet strain spike gene from HC/SZ/61/03 (Rockx et al. 2007). An infectious clone harboring the clinical isolate GZ02 or civet HC/SZ/61/03 spikes exhibited severe weight loss and death, as well as pathology consistent with acute respiratory distress syndrome in aged, but not young mice (Rockx et al. 2007). Despite these early achievements with aged mice for SARS-CoV mouse model development, it is impractical to maintain large colonies of aged mice; and immune senescence in aged mice may complicate studies involving pathogenesis and effective evaluation of immune responses to therapeutic countermeasures. In addition to these limitations, full-length clinical isolates of SARS-CoV were not shown to be lethal in aged mice, a clinical outcome that impacted nearly 10% of all human cases.

Lethality was not achievable using clinical SARS-CoV isolates in various young wild-type or immune-incompetent mouse strains. With this in mind, attention turned to genetic modification of mice to acquire a lethal model for SARS-CoV pathogenesis. In order to continue working with unaltered human clinical isolates of SARS-CoV, mouse strains constitutively expressing the human receptor for SARS-CoV, human angiotensin converting enzyme 2 (hACE2), were generated. The logic followed that human clinical isolates of SARS-CoV are evolved to use the hACE2 receptor more effectively than the innate mouse ACE2 (mACE2) receptor; therefore, may elicit pathology consistent with a lethal respiratory infection. Different constitutive promotors were used to express hACE2 in mice: cytokeratin promotor (McCray et al. 2007; Netland et al. 2008), chicken beta-actin promotor with a cytomegalovirus IE enhancer (Tseng et al. 2007), and the mouse ACE2 promotor (Yang et al. 2007). Expression levels of human ACE2 correlated with disease severity in all transgenic mouse models. Human ACE2 overexpression mice generated with the cytokeratin promoter and the mACE2 promoter were attempts to limit expression to respiratory cells and cells that exhibited innate mACE2 receptor expression, respectively (McCray et al. 2007; Netland et al. 2008; Yang et al. 2007). Even though these transgenic mice showed infection of airway epithelium, all of them exhibited high levels of hACE2 expression in the brains of transgenic mice which supported increased viral load in brain tissue (McCray et al. 2007; Netland et al. 2008; Tseng et al. 2007; Yang et al. 2007). The increased viral loads in the brains of infected animals ultimately led to mortality caused by extensive dissemination and encephalitis in the brain (McCray et al. 2007; Netland et al. 2008; Tseng et al. 2007; Yang et al. 2007). Accordingly, despite the successful generation of lethal hACE2 overexpression mouse models for SARS-CoV pathogenesis, neurological-related mortality confounded their value as models that could effectively mimic lethal respiratory disease often observed in infected humans.

Adaptive experimental evolution has been a staple of virology for > 60 years (Kalter 1949), providing critical insights into pathogenic mechanisms while bestowing robust, lethal mouse models to interrogate the efficacy of vaccines and therapeutics (Bolles et al. 2011; Cockrell et al. 2016; Rasmussen et al. 2014; Roberts et al. 2007a). Adaptive experimental evolution is performed by serial in vivo passages in the tissue of interest to adapt the virus to the host innate and adaptive immune responses. This is similar to the ongoing battle between a virus and the host immune responses occurring in nature, but on an expedited time-scale and in a tissue-specific manner. Tissue-specific adaptive evolution places the virus under selective pressure for mutations that allow for more efficient replication. To adapt SARS-CoV to cause severe acute respiratory disease in mouse lungs, 6-week-old female BALB/c mice were intra-nasally infected with the clinical Urbani isolate (Roberts et al. 2007a). This process was repeated (15 rounds) until signs of clinical disease were observed, predominantly weight loss that reached clinical endpoints for humane euthanasia (considered humane lethality) (Roberts et al. 2007a). The resulting mouse-adapted SARS-CoV was designated MA15. Rapid weight loss was accompanied by high viral titer in the lungs, viremia and dissemination to extrapulmonary sites, lymphopenia, neutrophilia, and histopathological changes in the lung commonly associated with pneumonitis. Accordingly, mortality was a consequence of extensive viral replication which led to virus-associated destruction of pneumocytes and epithelial cells. Only six coding mutations were sufficient to render the SARS-CoV Urbani human clinical isolate 100% lethal in 4 weeks old, 6–8 weeks old, and aged BALB/c mice (Roberts et al. 2007a). The MA15 SARS-CoV has since been used in a multitude of studies and proven to not only cause lethal disease in BALB/c mice but also in other mouse strains (Deng et al. 2014; Frieman et al. 2010; Gralinski et al. 2015, 2017; Totura et al. 2015; Zhao et al. 2012). Additional passaging of MA15 resulted in the generation of other mouse-adapted SARS-CoV strains including MA20 (20 passages) (Frieman et al. 2012), while an independent research group acquired a lethal SARS-CoV model by passaging the human Urbani isolate 25 rounds through the lungs of BALB/c mice, resulting in v2163 (Day et al. 2009). In addition to acquiring lethal SARS-CoV models of disease, comparing various passages of mouse-adapted SARS-CoV provides the unique advantage of being able to identify which SARS-CoV proteins acquire specific mutations that can elicit severe respiratory phenotypes. Identification of adaptive mutations acquired in specific SARS-CoV proteins provided insight into virus–host interaction networks that may be used for the development of virus-directed therapeutic countermeasures. Combining mouse-adapted SARS-CoV strains with mouse models that support interrogation of the host genome for molecules that influence SARS-CoV pathogenesis can reveal import interaction nodes amenable to host-directed therapeutic intervention.

As mentioned above, a number of early SARS-CoV mouse model studies showed that disease progression and outcome are mouse strain dependent, indicating that host genetics have a considerable influence on SARS-CoV pathogenesis. This is not surprising since human gene association studies have indicated that differences in an individual’s genetics may govern susceptibility and clinical outcomes of respiratory viral pathogenesis (Everitt et al. 2012; Forton et al. 2009; Kenney et al. 2017; Mills et al. 2014; Pasanen et al. 2017; Patarcic et al. 2015; Zhou et al. 2012), including retrospective studies of SARS-CoV-infected patients (Chan et al. 2007, 2006; Ching et al. 2010; Wang et al. 2008; Zhu et al. 2011). However, human genetic associations do not indicate cause-and-effect, which require the capacity to model the impact of host genetics on respiratory viral pathogenesis, in this case SARS-CoV pathogenesis. A recently developed, innovative resource for genetic mapping, called the Collaborative Cross (CC), comprises a panel of recombinant inbred mouse strains containing tractable genetic diversity that approaches the genetic diversity in the human population (Churchill et al. 2004; Threadgill et al. 2002). Using an octo-parental breeding scheme that includes classical laboratory stains (A/J, C57BL/6J, and 129/SvImJ), mouse models for human diseases (NOD/ShiLtJ for diabetes; NZO/HlLtJ for obesity), and wild-derived mouse strains (CAST/EiJ, PWK/PhJ, and WSB/EiJ), the CC captures 90% of the genetic variation present in the three major mouse subspecies (*Mus musculus musculus, Mus musculus domesticus, Mus musculus castaneus*) (Roberts et al. 2007b). Virus infection studies in CC mouse lines, including SARS-CoV, have led to mapping of high and low host response alleles as they relate to development of clinical signs of disease following viral pathogenesis (Bottomly et al. 2012; Brinkmeyer-Langford et al. 2017; Ferris et al. 2013; Gralinski et al. 2015, 2017; Green et al. 2017; Rasmussen et al. 2014). Rapid genetic mapping is most successful through comparative analysis of CC strains that show extreme phenotypes such as high versus low weight loss, or high versus low lung titers. Extreme phenotypes in select CC strains may reflect clinical outcomes observed in human disease, as observed in testing mouse-adapted Ebola virus in different CC strains (Rasmussen et al. 2014). Applying the CC technology to SARS-CoV pathogenesis identified two CC strains (CC003/Unc and CC053/Unc) with opposing susceptibility profiles (Gralinski et al. 2017). Genetic mapping revealed an adaptor protein (Ticam2) in the toll-like receptor pathway as a strong candidate driving severe respiratory disease phenotypes (Gralinski et al. 2017). Based on these observations, the CC mouse platform can be used to identify novel mouse models that recapitulate human clinical outcomes resulting from pathogenic viruses.

The CC mouse strains are an extraordinarily powerful platform to establish novel mouse models for emergent and pre-emergent isolates of respiratory coronaviruses. Harnessing the power of CC host genetic mapping will support the identification of novel hCoV–host molecular interaction networks that can be subsequently validated in genetically engineered mice. Innovations in gene editing technologies such as CRISPR/Cas9, TALENs, and synthetic zinc fingers have augmented the efficiency of genetic engineering in mice, making genomic modifications (i.e., allele-specific mutations, allele swaps, knock-outs, knock-ins) feasible in multiple mouse species, on a large-scale (Doench 2018; Kim and Kim 2014). Coincidentally, advances in the CRISPR/Cas9 technology overlapped with the outbreak of MERS-CoV in 2012, which was fortuitous for the development of lethal MERS-CoV mouse models.

### Mouse models for MERS-CoV pathogenesis

Building on expertise from the SARS-CoV outbreak, coronavirus researchers immediately recognized the overwhelming need for an effective MERS-CoV mouse model following the emergence of MERS-CoV in 2012. However, researchers were perplexed to find that mouse lines conventionally susceptible to SARS-CoV infection/replication were completely resistant to infection with clinical MERS-CoV isolates (Coleman et al. 2014). Initial studies demonstrated that non-human primates were susceptible to clinical isolates of MERS-CoV (de Wit et al. 2013b; Falzarano et al. 2014; Johnson et al. 2016, 2015; Munster et al. 2013); therefore, the inability to infect mice with MERS-CoV was likely not due to restriction by the host immune responses. This was validated in immune-incompetent mouse lines that also lacked the capacity for infection/replication of MERS-CoV (Coleman et al. 2014). Concurrent with some of the initial MERS-CoV studies in mice, Raj et al. identified a novel receptor for MERS-CoV, human dipeptidyl peptidase 4 (hDPP4) (Raj et al. 2013). Unlike SARS-CoV which could infect mice through interaction of the RBD in the spike protein with the mACE2 receptor, mouse DPP4 (mDPP4) did not support an interaction with the MERS-CoV spike protein (Cockrell et al. 2014). Crystal structures of the MERS-CoV spike-hDPP4 interface revealed a number of specific amino acids necessary for an interaction between the RBD in spike and two specific domains, referred to as blades IV and V on the β-propeller structure, of the hDPP4 structure (Lu et al. 2013; Wang et al. 2013). Ectopic expression studies with the mouse DPP4 receptor revealed that altering a minimum of two amino acids on the mDPP4 (positions A288 and T330) conferred susceptibility of the human fibroblast cell line, 293T, to MERS-CoV infection (Cockrell et al. 2014; Peck et al. 2015). Importantly, in mice T330 is an N-linked glycosylation site that may sterically hinder MERS-CoV infection, and is not present in DPP4 orthologues from susceptible species including human, NHPs, bats, and camels (Peck et al. 2015). Apparently, ferret and hamster DPP4 may also have similar glycosylation’s, which may explain the inability of MERS-CoV to also infect/replicate in these conventional small animal models (de Wit et al. 2013a; Peck et al. 2017; Raj et al. 2014; van Doremalen et al. 2014). Species-specific challenges utilizing the DPP4 receptor not only limited development of mouse models, but stymied development of all small animal models required for evaluating therapeutic countermeasures.

**Fig. 3:**
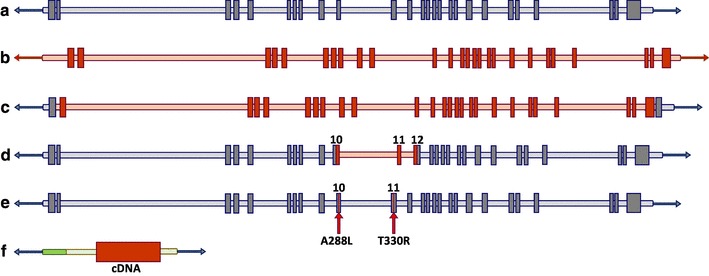
Comparison of mouse models for MERS-CoV. **a** Wild-type mouse DPP4 (mDPP4) with 26 exons (NM_010074.3). **b** Wild-type human DPP4 (hDPP4) with 26 exons (NM_001935.3). **c** DPP4 sequence used in VelociGene hDPP4 knock-in mouse model (Pascal et al. 2015). Mouse genomic sequence, from exon 2 through the stop codon in exon 26, was deleted and replaced with exon 2 through exon 26 and a portion of 3′ untranslated sequence of human genomic sequence. **d** DPP4 sequence used in hDPP4 knock-in model (Li et al. 2017). Mouse genomic sequence from codon I264 in exon 10 to codon V340 in exon 12 was replaced with the human equivalent. **e** DPP4 sequence used in 288–330^+/+^ mouse model (Cockrell et al. 2016). CRISPR/Cas9 technology used to make A288L substitution in exon 10 and T330R substitution in exon 11 of mouse DPP4. **f** DPP4 construct used in hDPP4 overexpression mouse models. Constructs included cDNA from human DPP4 flanked by constitutive promoter, polyadenylation signals, and other regulatory elements (Agrawal et al. 2015); or by 5′ and 3′ genomic regions of human cytokeratin 18 or human surfactant protein C (Li et al. 2016)

Accordingly, the initial development of mouse models for MERS-CoV required employing a number of methods that focused on engineering mice to express/overexpress the human DPP4 receptor (Agrawal et al. 2015; Li et al. 2016; Zhao et al. 2015, 2014) (Fig. 3). The first mouse model, developed by Zhao et al., established transient expression of human DPP4 in the airway of BALB/c mice, C57BL/6 mice, and several knock-out mice using an adenoviral vector to transduce an hDPP4 overexpression cassette (Ad-hDPP4) to the lungs of mice (Zhao et al. 2014). Transient hDPP4 expression rendered mice susceptible to clinical isolates of MERS-CoV infection [1 × 10^5^ plaque forming units (PFU) dose], supporting viral replication in the lungs and producing transient weight loss with mild pneumonia, particularly in older and immunodeficient mice (Zhao et al. 2014). No mortality or signs of advanced clinical respiratory disease were observed (Zhao et al. 2014). As a platform strategy that could be applied to novel emerging viruses, the Ad-hDPP4 model provided a means to rapidly evaluate MERS-CoV-directed therapeutic countermeasures with regard to interfering with replication in the lungs of infected mice. Nevertheless, the Ad-hDPP4 model was not an effective means of understanding how therapeutic countermeasures could prevent development of severe respiratory disease that resulted in ~ 35% mortality in infected human cases.

To develop more clinically relevant mouse models, a number of groups turned to more conventional hDPP4 knock-in approaches, resulting in ubiquitous, constitutive overexpression of hDPP4 throughout the mouse (Fig. 3). In 2015, Agrawal et al. reported development of a robust MERS-CoV mouse model by placing expression of hDPP4 under the CAG promoter (Agrawal et al. 2015). Infection with clinical isolates of MERS-CoV resulted in high viral loads in the lungs that produced severe respiratory disease and was fatal by day 6 post-infection (Agrawal et al. 2015). However, disease was confounded by high viral loads in extrapulmonary tissues including the brain, heart, spleen, kidney, and intestines (Agrawal et al. 2015). In an independent study, Zhao et al. demonstrated multi-organ damage in an hDPP4 overexpression mouse model employing the same promoter (Zhao et al. 2015). The MERS-CoV viral loads were nearly two logs higher in the brains of infected mice than in the lungs and corresponded to viral encephalitis observed in the brain by day 9, with mice exhibiting signs of paralysis (Zhao et al. 2015). Li et al. went a step further by generating hDPP4 overexpression models with the goal of restricting expression to epithelial cells (Li et al. 2016). Two different models were generated using either the cytokeratin 18 (K18) or surfactant C protein (SPC) promoter to drive expression of hDPP4. Mouse lines derived from the SPC-hDPP4 cassette did not exhibit mortality or signs of respiratory disease. In contrast, infection of K18-hDPP4 mice with 1 × 10^5^ PFU of a MERS-CoV clinical isolate (EMC_2012) produced weight loss, lung hemorrhaging and mortality by 6–7 days p.i., and lung pathology consistent with severe respiratory disease (Li et al. 2016). Unfortunately, the K18 promoter did not limit MERS-CoV infection to the lungs, instead high viral loads in the brains of infected mice caused neurological disease that was consistent with the observed mortality (Li et al. 2016).

In a novel genetic engineering approach, Pascal et al. employed Regeneron’s VelociGene technology to precisely replace the entire *mDPP4* coding region (~ 79 kb) with the *hDPP4* gene, which included ~ 82 kb of sequence encoding both introns and exons (Pascal et al. 2015) (Fig. 3). Despite removal of nearly the entire *mDPP4* gene, the 5′ and 3′ untranslated regions of *mDPP4* were maintained; thereby, retaining the endogenous mDPP4 promoter and RNA termination regions conferred expression levels and tissue distribution for hDPP4 similar to that obtained for mDPP4. Infection with 2 × 10^5^ PFU of a MERS-CoV clinical isolate resulted in viral replication in the lungs with mild clinical disease and pathology indicative of inflammation, with no weight loss or mortality through day 4 p.i. (Pascal et al. 2015). Subsequent studies with the VelociGene mice comprised a more detailed evaluation of the model, wherein mice infected with 2.5 × 10^4^ PFU achieved dramatic weight loss and mortality (using 20% cut-off) by 7 days p.i. (Coleman et al. 2017). Lung pathology indicated that mice developed moderate signs of respiratory infection with little indication of severe respiratory disease. These studies were also the first indication that CD8^+^ T cells and macrophages play a significant role in MERS-CoV pathogenesis (Coleman et al. 2017). Importantly, MERS-CoV infection/replication was primarily in the lungs, with little involvement of extrapulmonary tissues (Coleman et al. 2017; Pascal et al. 2015). The lack of brain lesions in this model was a significant advancement over transgenic models that relied on global/ubiquitous hDPP4 expression. Nonetheless, this model is not readily available to the scientific community by Regeneron, and if the model is obtained it comes with a number of commercial restrictions from Regeneron that limit its utility when considering development of therapeutic countermeasures (declaration by the authors of this manuscript).

Another limitation often overlooked with hDPP4 expression models is the innate functions of DPP4 that have evolved in a species-specific manner to maintain various physiological processes inherent to the host. Altered DPP4 activity and/or expression is associated with many pathological conditions that include psycho-neuroendocrine disorders, solid tumor cancers, hematological malignancies, infectious disease, and autoimmune/inflammatory diseases (Klemann et al. 2016). The enormous biological breadth of DPP4 is predominantly through its enzymatic activity wherein it cleaves off the amino terminal dipeptides of various biological substrates having l-alanine or l-proline at the penultimate position (Klemann et al. 2016). One of the more important physiological processes of DPP4, also known as CD26, is the modification of numerous cytokine and chemokines involved in the maintenance of immunological homeostasis (Klemann et al. 2016; Ohnuma et al. 2008). As a cell surface molecule on CD4^+^ and CD8^+^ T cells, DPP4 influences numerous immunological processes including T cell activation and proliferation, cytokine production, differentiation to immunoglobulin producing plasma cells, and transendothelial migration (Ohnuma et al. 2008). Therefore, overexpressing full-length hDPP4 or replacing mDPP4 with full-length hDPP4 in the knock-in mouse models may significantly alter the inherent biological and immunological processes that mDPP4 has evolved to execute in a species-specific manner, in the mouse. Ultimately, if hDPP4 expression results in a modified immune response in mice, these could artificially influence the pathogenic outcomes of MERS-CoV infections and immunological responses that are central to evaluating the efficacy of therapeutic countermeasures.

The goal of the most recent generation of mouse models was to genetically modify the mDPP4 receptor so that it would be amenable to interaction with the MERS-CoV spike RBD and subsequent infection, thereby avoiding the need to introduce the hDPP4 receptor. Achieving a MERS-CoV susceptible mDPP4 with minimal modifications would be central to limiting any functional alteration to mDPP4 that could interfere with its broad influence over multiple host physiological processes. In 2016, Cockrell et al. utilized the CRISPR/Cas9 technology to make two amino acid substitutions in the mDPP4 gene (A288L and T330R) of C57BL/6J mice (Cockrell et al. 2016) (Fig. 3). As discussed above, in vitro overexpression of the modified mDPP4 was previously found to alter the susceptibility of cell lines to MERS-CoV infection (Cockrell et al. 2014; Peck et al. 2015). Mice with the modified DPP4 (referred to as 288–330^+/+^ mice) encode both amino acid substitutions on both chromosomes. Characterization of this model demonstrated that innate mDPP4 expression profiles and physiological processes influenced by DPP4 (glucose metabolism and T cell activation) were not altered by the two amino acid modifications of mDPP4. The 288–330^+/+^ mice infected with various isolates of MERS-CoV (clinical isolates, a camel isolate, and molecular clones) supported high viral replication in the lungs, but failed to exhibit clinical signs of disease (Cockrell et al. 2016). Adaptive evolution through 15 rounds of serial passage in 288–330^+/−^ mice yielded a mouse-adapted MERS-CoV capable of achieving lethality, decreased respiratory function, and lung pathology indicative of severe acute respiratory distress syndrome at viral doses of 5 × 10^6^ PFU (Cockrell et al. 2016). MERS-CoV infected 288–330^+/+^ mice that exhibited severe respiratory disease were absent of any detectable virus in the brain. The development of severe respiratory disease and mortality could be prevented using a MERS-CoV spike-directed vaccine, and through prophylactic administration of an antibody therapeutic (Cockrell et al. 2016). Although the 288–330^+/+^ model is highly effective for studying MERS-CoV pathogenesis and evaluating therapeutic countermeasures, the high infectious dose (5 × 10^6^ PFU) required to achieve severe respiratory disease and mortality left room for improvement. An additional 20 rounds of passaging resulted in a mouse-adapted virus capable of achieving similar severe respiratory disease and mortality, but at substantially lower doses (10^3^–10^5^ PFU) (Douglas et al. 2018). Overall, limiting changes in the mDPP4 receptor to two amino acids, researchers were able to minimize disruption of the natural expression levels, distribution, and biological functions of mDPP4. The 288–330^+/+^ MERS-CoV mouse model continues to have a central role in studies investigating MERS-CoV pathogenesis and therapeutic countermeasures (Menachery et al. 2017b, c).

The most recent mouse model consists of a genetically modified mDPP4 receptor through replacement of exons 10–12 (including introns) of the *mDPP4* locus with the *hDPP4* equivalent, resulting in a minimal knock-in mouse model referred to as hDPP4-KI (Li et al. 2017) (Fig. 3). Modification of mDPP4 in the hDPP4-KI model is much smaller than the Regeneron full-length hDPP4 knock-in model, but larger than the two amino acid modifications to mDPP4 in the 288–330^+/+^ model, placing it somewhere between the two types of models. Regarding biological and immunological function, it is not clear if the modified mDPP4 in the hDPP4-KI model has altered expression, bio-distribution, or functional profiles compared to wild-type mDPP4 receptor. Nevertheless, the hDPP4-KI mice were susceptible to infection with clinical isolates of MERS-CoV, resulting in high levels of viral replication in the lungs but no signs of respiratory disease. A mouse-adapted MERS-CoV was achieved through 31 serial passages of MERS-CoV, inducing severe respiratory disease accompanied by high mortality at infectious doses of 10^4^–10^5^ PFU (Li et al. 2017). Similar to the 288–330^+/+^ model, the hDPP4-KI model exhibited little involvement of extrapulmonary tissues in MERS-CoV pathogenesis (Li et al. 2017). The mouse-adapted viruses acquired by Li et al. revealed unique mutations in specific viral proteins that may be critical for achieving and evading host immune responses. Comparison of these viruses to those obtained by Douglas et al. demonstrated that changes at amino acid 222 in the MERS-CoV spike protein may be critical to achieve lethal infection at low infectious doses (Douglas et al. 2018; Li et al. 2017). Importantly, this mutation was not present in earlier passages by Cockrell et al. in the 288–330^+/+^ model, which required high infectious doses to achieve severe respiratory disease and lethality (Cockrell et al. 2016). Mouse-adapted viruses, such as those used by Cockrell et al., Douglas et al. and Li et al. can help provide insight into adaptive evolution and identify specific mutations or regions of the virus that are important for enhancing virus fidelity in the host and/or evading the host immune responses.

Adaptations in MERS-CoV most notably occur in the spike region, which is an important determinant of host tropism. Adapted MERS-CoV strains with mutations in this region may not provide an accurate representation of the observed viral pathogenesis in humans. Currently, achieving lethal disease in mice with clinical isolates of MERS-CoV require expression of full-length hDPP4, as demonstrated for the Regeneron hDPP4 knock-in mice. However, altering DPP4 significantly through overexpression or replacement of mDPP4 can disrupt the immunological homeostasis of the animal, making it difficult to analyze disease etiology and immune responses to therapeutic countermeasures. A MERS-CoV mouse model would ideally involve infection with clinical MERS-CoV isolates in an unmodified mouse. One possibility is utilizing CC mice, as described above for SARS-CoV, which offer a wider range of genetic variation than the genetically engineered C57BL/6 mice used in currently established mouse models. However, unlike SARS-CoV, the CC mice are not susceptible to MERS-CoV. Crossing various CC lines with mice that have minimal alterations to mDPP4 (e.g., 288–330^+/+^ mice), or direct genetic engineering of CC lines using CRISPR/Cas9 technologies, can produce mouse lines that maintain native mouse DPP4 expression, distribution, and functional profiles, but can be screened for differential susceptibility to clinical isolates of MERS-CoV. The capacity to genetically engineer receptors to alter mouse susceptibility to hCoV infection, combined with adaptive hCoV evolution and genetically diverse CC mouse strains, establishes a platform that can be used to evaluate the pathogenesis of pre-emergent hCoVs with unknown etiologies.

### Identifying pre-emergent hCoVs with pathogenic potential

The mouse tools founded from studies of SARS-CoV and MERS-CoV pathogenesis will aid in understanding if specific pre-emergent CoVs have pathogenic potential in mammalian models of severe respiratory disease. Most hCoVs (SARS, MERS, 229E, and NL63) are considered to have their origin in bats [reviewed in (Forni et al. 2017; Menachery et al. 2017a)]. Metagenomics analysis of various bat species has identified a diverse repertoire of SARS-like (SL-bCoVs) and MERS-like (ML-bCoVs) bat coronaviruses (Ge et al. 2012, 2013; He et al. 2014; Lau et al. 2015, 2010, 2005; Wu et al. 2016; Yang et al. 2016). Those with pre-emergent potential include SL-CoVs WIV1 and WIV16, shown to utilize the ACE2 receptor (Ge et al. 2013; Menachery et al. 2015; Yang et al. 2016), and an ML-CoV HKU4, demonstrated to utilize the DPP4 receptor for infection (Wang et al. 2014; Yang et al. 2014). Notably, WIV1 and WIV16 are the only pre-emergent CoVs directly isolated from bat feces on Vero E6 cells and shown to replicate in various cell lines expressing the human, or NHP, ACE2 receptor (Ge et al. 2013; Yang et al. 2016). In contrast to WIV1 and WIV16, isolated HKU4 was not demonstrated to infect cells, but rather lentiviral particles pseudotyped with HKU4 spike protein supported efficient binding and infection of cells expressing hDPP4 (Wang et al. 2014; Yang et al. 2014). The structure of CoV spike proteins in the context of pseudotyped lentiviral particles may differ to that in CoV particles, and may account for the difficulty of HKU4 infecting and replicating on cells expressing hDPP4.

The spike proteins of many pre-emergent bat CoVs (bCoVs) have amino acid differences that preclude efficient interaction with known human host receptors, such as hACE2 or mACE2. Transmission to intermediate hosts (civets or camels) and humans may require additional adaptations in the spike protein [reviewed in (Graham and Baric 2010; Menachery et al. 2017a)]. Therefore, to evaluate pathogenesis of pre-emergent bCoVs in mouse models, the spike proteins were replaced by generating infectious molecular clones with known spike proteins from clinical isolates of SARS or mouse-adapted SARS-CoV (Agnihothram et al. 2014; Becker et al. 2008; Menachery et al. 2015, 2016). The first of these studies derived an infectious molecular clone SL-bCoV from sequences of four previously identified SL-bCoVs (HKU3-1, HKU3-2, HKU3-3, and RP3) (Becker et al. 2008). The chimeric virus was able to replicate in BALB/c mouse lungs, but did not elicit signs of respiratory disease commonly associated with SARS-CoV (Becker et al. 2008). Similarly, a chimeric WIV1 (WIV1-MA15) replicated in BALB/c mouse lungs, but was attenuated with regard to causing respiratory disease (Menachery et al. 2016). Attenuation of WIV1-MA15 was partially overcome by infecting HFH4 mice that overexpress the hACE2 receptor (Menachery et al. 2016), indicating that mouse adaptation of the WIV1-MA15 to mACE2 could enhance disease in wild-type BALB/c mice. Indeed, Agnihothram et al. demonstrated that mouse adaptation of an infectious molecular clone of an HKU5-SE chimeric resulted in dramatic respiratory disease in aged BALB/c mice (Agnihothram et al. 2014). HKU5 is closely related to MERS-CoV, but since there was no mouse model available for MERS-CoV at the time, it was more practical to replace the HKU5 spike with the mouse-adapted SARS-CoV spike. These studies demonstrated that adaptive mutations outside of the spike are necessary to elicit respiratory disease in different mammalian species. However, this was not the case for the SHC014 bCoV chimeric virus (SHC014-MA15), which caused significant weight loss in BALB/c mice and replicated to titers comparable to SARS-CoV MA15 in the lungs (Menachery et al. 2015). Moreover, wild-type SHC014 spike protein supported infection/replication in the lungs of BALB/c mice, but did not result in disease. Applying the classical approach of adapting SHC014 to mice by serial passaging may be beneficial, but can be time-consuming. Screening SHC014, or other pre-emergent bCoVs, in CC mouse strains has the potential to rapidly lead to mouse models capturing different, or even all, aspects of clinical disease in human patients.

### Evaluating therapeutic countermeasures in hCoV mouse models

The ultimate goal for mouse model development is capturing disease etiologies that closely reflect what is observed during human CoV infection, so the efficacy of various therapeutic countermeasures can be evaluated for effective resolution of pathogenic outcomes. A number of hCoV-directed drug, antibody, and vaccine therapeutics were evaluated for efficacy against SARS-CoV until 2012 when the focus of therapeutic development shifted to MERS-CoV [reviewed in (Dyall et al. 2017; Zumla et al. 2016)]. Currently, there are no FDA-approved therapeutics for the specific treatment of SARS-CoV or MERS-CoV. Various combinations/types of broad spectrum antivirals (e.g., ribavirin) combined with interferon therapies (interferon α or β), and/or immune modulators (e.g., corticosteroids) were ineffective for SARS-CoV and MERS-CoV, and in some cases appeared to worsen disease [reviewed in (Dyall et al. 2017; Zumla et al. 2016)]. A recent clinical trial seeks to interfere with MERS-CoV replication using a combination therapy that includes FDA-approved protease inhibitors (Lopinavir/ritonavir) (https://clinicaltrials.gov/ct2/show/study/NCT02845843). Repurposing FDA-approved drugs has been the interest of numerous therapeutic studies, but little is known regarding the efficacy of drug therapeutics against SARS-CoV or MERS-CoV outside of cell-based in vitro studies [reviewed in (Dyall et al. 2017)]. Mouse models confer distinct advantages for therapeutic examination including cost savings through small scale production for testing, and experimental reproducibility.

The recent development of a novel nucleotide prodrug, GS-5734, demonstrated efficacy against Ebola virus in NHPs (Warren et al. 2016), and has since moved into phase 2 clinical trials for Ebola [https://clinicaltrials.gov/ct2/show/NCT02818582?cond=PREVAIL+IV&rank=1; (Dornemann et al. 2017)]. A recent study demonstrated broad spectrum efficacy of GS-5734 against multiple hCoVs (SARS-CoV, MERS-CoV, and NL63) and pre-emergent bCoVs (HKU3, HKU5, SCH014, and WIV1) on primary human airway epithelial cells (Sheahan et al. 2017). Importantly, GS-5734 exhibited in vivo efficacy against SARS-CoV-induced immunopathology if the drug was administered prophylactically, or a therapeutic dose 24-h post-infection (Sheahan et al. 2017). Delaying treatment until 48-h post-infection did not ameliorate disease symptoms. Although the kinetics of SARS-CoV pathogenesis will differ between mouse and human, these results indicate that the molecular programs leading to respiratory immunopathology are established shortly after infection, thereby limiting effective therapeutic treatment to a short window, post-infection. The study by Sheahan et al. also demonstrates the importance of having mouse models that recapitulate severe respiratory disease often seen in humans (Sheahan et al. 2017). An effective therapeutic should not only prevent viral replication, but more importantly should effectively restrict respiratory pathogenesis. Achieving both may require therapeutic intervention with a combination of an hCoV antiviral and a host-directed therapy that curtails immunopathology associated with acute respiratory distress syndrome. Only in the last 3 years have MERS-CoV mouse models, described above, become available to assess therapeutics; therefore, future studies will include evaluating in vivo efficacy of GS-5734 against MERS-CoV in the 288–330^+/+^ model described above.

As an ongoing threat to worldwide public health, a plethora of therapeutic research focused on development of MERS-CoV-specific antibodies and vaccines, both of which have been extensively reviewed [reviewed in (Arabi et al. 2017; Dyall et al. 2017; Okba et al. 2017)]. Importantly, few studies have evaluated the therapeutic efficacy of MERS-CoV antibodies and vaccines in mammalian challenge models that elicit severe respiratory disease, often resulting in death (Cockrell et al. 2016; Munster et al. 2017; Tai et al. 2016; van Doremalen et al. 2017; Wang et al. 2018). Rather, many preclinical studies exhibited efficacy in the mouse or NHP challenge models with protection defined as reduced viral loads in the lungs by plaque assay or RT-PCR. Preclinical results have expedited the process of moving some antibody and vaccine therapeutics into early clinical trials [reviewed in (Arabi et al. 2017; Dyall et al. 2017; Okba et al. 2017)]. Based on results obtained in studies with GS-5734, it will be critical to determine efficacy of antibody and vaccine therapeutics in lethal respiratory models of MERS-CoV infection.

Vaccine countermeasures are not only being considered as intervention strategies for staving off disease in humans, but have also exhibited efficacy in dromedary camels, the MERS-CoV zoonotic host (Haagmans et al. 2016; Muthumani et al. 2015). Targeting zoonotic hosts, such as dromedary camels or bat populations, may be an effective means of curtailing transmission into the human population. Thinking beyond vaccine countermeasures, we now have the ability to harness genome editing technologies that could allow us to intervene with the process of zoonotic transmission. C57BL/6J mice were made susceptible to MERS-CoV by using CRISPR/Cas9 technologies to modify two amino acids at positions 288 and 330 on mouse DPP4, to the orthologous amino acids encoded by human DPP4 (Cockrell et al. 2016). Genetic modification of the human DPP4 orthologues in camel and bat to look like mouse DPP4 at these positions may result in camels and bats that are now resistant to MERS-CoV infection. Although introduction of MERS-CoV-resistant bats into the environment may take a long time, camels are considered farm animals in many parts of Northern Africa and the Middle East with controlled breeding practices; therefore, it may be feasible to establish resistant herds that are unable to transmit MERS-CoV to humans. Genome editing of farm animals is clearly achievable as recently demonstrated in pigs modified with CRISPR/Cas9 for genome-wide inactivation of porcine endogenous retroviruses (PERVs), thereby eliminating the potential of cross-species transmission of PERVs during xenotransplantation of pig organs into humans, with the intention of easing the shortage of available human organs (Niu et al. 2017; Yang et al. 2015).

## Conclusions

The combined power of genetic engineering of mice, reverse genetic systems for CoVs, mouse-adaptation, and genetically diverse mouse populations afford the tools to develop models that reproducibly recapitulate severe respiratory disease that can be caused by hCoVs in humans. Establishing mouse models for SARS-CoV and MERS-CoV have largely depended on one or more of these tools. Regardless of how effective models for SARS-CoV and MERS-CoV are for studying pathogenesis and therapeutic interventions, there are drawbacks related to mouse-adapted and transgenic mouse models that alter susceptibility and pathogenesis to hCoVs. Acquired mutations associated with severe respiratory disease in mouse-adapted hCoVs may have no bearing on human pathogenesis or have altered pathogenic outcomes in humans. Genetically engineering a specific host species to enhance susceptibility restricts applications of the mouse model, requiring cross-breeding to introduce novel host mutations from knock-out mouse lines and CC mouse strains. This process is labor intensive and requires an extensive timeline. Alternatively, one could introduce mutations directly into genetically diverse lines; however, this is not cost effective. Additionally, it is difficult to replicate and study co-morbidities in mice that are associated with lethal respiratory disease seen in humans. Pre-existing conditions such as advanced age, diabetes, chronic lung diseases, heart disease, and kidney disease have been reported in many of the severe/lethal MERS-CoV human cases [reviewed in (Arabi et al. 2017)]. One way to address many of these concerns could be to screen genetically diverse populations of mice (e.g., Collaborative Cross) with clinical isolates or infectious molecular clones of wild-type strains from emergent, or pre-emergent, hCoVs. Screening in genetically diverse mouse populations may yield mouse models with a range of clinically relevant phenotypes that more closely reflect the plethora of respiratory disease outcomes observed in the human population. Experiences from SARS-CoV and MERS-CoV have taught researchers that each hCoV may require unique approaches for mouse model development. The mouse tools instituted for SARS-CoV and MERS-CoV constitute a versatile preclinical platform for addressing global pathogen preparedness, a directive of the World Health Organization.
